# Gene expression profiling in the intestinal mucosa of obese rats administered probiotic bacteria

**DOI:** 10.1038/sdata.2017.186

**Published:** 2017-12-12

**Authors:** Julio Plaza-Díaz, Cándido Robles-Sánchez, Francisco Abadía-Molina, María José Sáez-Lara, Laura María Vilchez-Padial, Ángel Gil, Carolina Gómez-Llorente, Luis Fontana

**Affiliations:** 1 Department of Biochemistry and Molecular Biology II, School of Pharmacy, University of Granada, Granada 18071, Spain; 2 Institute of Nutrition and Food Technology ‘José Mataix’, Biomedical Research Center, Parque Tecnológico Ciencias de la Salud, University of Granada, Granada 18016, Spain; 3 Instituto de Investigación Biosanitaria ibs.GRANADA, Spain; 4 Department of Cell Biology, School of Sciences, University of Granada, Granada 18071, Spain; 5 Department of Biochemistry and Molecular Biology I, School of Sciences, University of Granada, Granada 18071, Spain; 6 CIBEROBN, Instituto de Salud Carlos III, Madrid 28029, Spain

**Keywords:** Applied microbiology, Metabolic disorders

## Abstract

We investigated whether the administration of *Lactobacillus paracasei* CNCM I-4034, *Bifidobacterium breve* CNCM I-4035 and *Lactobacillus rhamnosus* CNCM I-4036 modulate the expression of genes in the intestinal mucosa of obese Zucker rats. Forty-eight Zucker-Lepr^*fa/fa*^ and 16 Zucker lean Lepr^+/*fa*^ rats were used. Eight Zucker lean Lepr^+/*fa*^ and 8 Zucker-Lepr^*fa/fa*^ rats were euthanized as a reference. The remaining 40 Zucker-Lepr^*fa/fa*^ rats were then assigned to receive 10^10^ colony forming units (CFU) of one of the three probiotic strains, a mixture of *L. paracasei* CNCM I-4034 and *B. breve* CNCM I-4035, or a placebo by oral administration for 30 days. An additional group of 8 Zucker lean Lepr^+/*fa*^ rats received the placebo for 30 days. Over 27,000 rat genes were studied using a DNA array. Four animals per group were used. Total RNA was extracted from intestinal mucosa and cDNA was synthesized, fragmented and labeled. Labeled cDNA was hybridized using GeneChip kits, and the latter were scanned. Intensity values of each probe were processed and normalized to obtain an individual value for each set of probes.

## Background & Summary

Metabolic syndrome, better referred to as insulin resistance syndrome (IRS), was originally defined as concomitant hyperlipidemia, hypertension, insulin resistance and obesity^1,2^. IRS often precedes the onset of type 2 diabetes and increases the risk of cardiovascular disease^3,4^. Accordingly, IRS has reached pandemic levels and become a major public health concern.

The Zucker rat shows many of the features of IRS; therefore, it is one of the most commonly used genetic models of this syndrome^4^. Zucker-Lepr^*fa/fa*^ rats exhibit obesity, hyperglycemia, insulin resistance, hypercholesterolemia, hypertriglyceridemia, and elevated serum free fatty acid concentrations in contrast to Zucker lean Lepr^*+/fa*^ rats. In addition, Zucker-Lepr^*fa/fa*^ rats have hepatic steatosis, as well as elevated serum aspartate aminotransferase (AST) and alanine aminotransferase (ALT) activities, indicating that the liver component of IRS is also present in this model^5^.

Probiotics are live microorganisms that, when consumed in adequate amounts, confer a health effect on the host^6^. Beneficial effects of probiotics have been reported in allergy, intestinal-related diseases, chronic liver disease, urinary tract infections and respiratory infections, among others^7^. Lactobacilli and bifidobacteria are the genera most frequently used as probiotics. A variety of mechanisms underlying their beneficial effects have been proposed: modification of the gut microbiota, competitive adherence to the mucosa and epithelium, strengthening of the gut epithelial barrier and modulation of the immune system to convey an advantage to the host^8^.

We have previously reported that the administration of three probiotic strains (*Lactobacillus paracasei* CNCM I-4034, *Bifidobacterium breve* CNCM I-4035 and *Lactobacillus rhamnosus* CNCM I-4036) to healthy human volunteers for 30 days is totally safe^9^ and that their administration for the same period of time to Zucker-Lepr*^fa/fa^* rats attenuates the accumulation of fat in the rats’ liver and exerts anti-inflammatory effects such as lower serum concentrations of tumor necrosis factor (TNF)-α, interleukin (IL)-6 and bacterial lipopolysaccharide (LPS)^5^. These three probiotic strains were isolated from the feces of breast-fed newborns. They were selected based on their *in vitro* properties such as adhesion to intestinal cells, sensitivity to antibiotics, and resistance to both acid pH and biliary salts. We have showed their safety in immunocompetent and immunodepressed mice, and that they inhibit *in vitro* the growth of *Listeria monocytogenes* as well as infections by human rotavirus^10^.

Some authors have described the modulation of gene expression by probiotics. Dykstra *et al.*^11^ reported the induction of the gene coding for mucin 3 (*Muc3*) in the small intestine of rats fed *Lactobacillus plantarum* 299v, *Lactobacillus rhamnosus* R0011, or *Bifidobacterium bifidum* R0071. Ohtsuka *et al.*^12^ administered *Bifidobacterium breve* M-16V to rat pups during the newborn period and found a lower expression of various inflammation-related genes in the colon. This descriptor is based on the data of our recently published work^13^, whose goal was to investigate whether these bacterial strains may modulate the gene expression of the intestinal mucosa. For this purpose and with the help of DNA microarray technology, we began by studying the modulation of a great number of genes in intestinal mucosa samples from obese Zucker rats. We found changes in expression of 1,501 genes due to the obese condition. The results of the array also showed changes in the expression of 40 genes for *L. paracasei* CNCM I-4034; 12 genes for *B. breve* CNCM I-4035; 24 genes for *L. rhamnosus* CNCM I-4036; and 3 genes for the mixture of *L. paracasei* CNCM I-4034 and *B. breve* CNCM I-4035. Expression of three genes (*Adamdec1*, *Ednrb* and *Ptgs1/Cox1*) was up-regulated in the intestinal mucosa of the obese rats compared with that in the rats when they were still lean. Probiotic administration down-regulated expression of *Adamdec1* and *Ednrb* at the mRNA and protein levels and that of *Ptgs1/Cox1* at the mRNA level, and this effect was in part mediated by a decrease in both macrophage and dendritic cell populations. Probiotic treatment also increased secretory IgA content and diminished the LPS-binding protein (LBP) concentration.

## Methods

These methods are expanded versions of descriptions in our related work^13^.

### Microorganisms

The probiotic strains *Lactobacillus paracasei* CNCM I-4034, *Bifidobacterium breve* CNCM I-4035, and *Lactobacillus rhamnosus* CNCM I-4036 have been characterized and are described elsewhere^10^. These strains were deposited in the *Collection Nationale de Cultures de Microorganismes* (CNCM) of the Institute Pasteur^10^.

### Ethical statement

This study was conducted in strict accordance with the recommendations in the guidelines for animal research of the University of Granada (Spain). All animals received humane care. The protocol was approved by the Committee on the Ethics of Animal Experiments of the University of Granada (Permit Number CEEA: 2011-377).

### Experimental design

**Figure 1:**
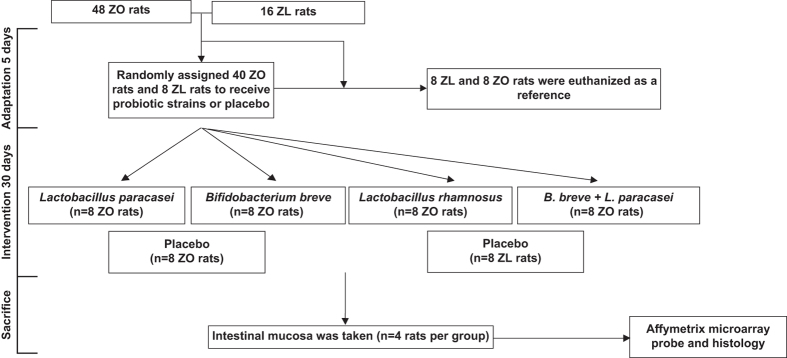
Workflow scheme. The number of rats from which high quality RNA was obtained is shown for each condition tested. Abbreviations: ZL, Zucker lean Lepr^+*/fa*^ rats; ZO, Zucker Lepr*^fa/fa^* rats.

Forty-eight Zucker-Lepr^*fa/fa*^ and 16 Zucker lean Lepr^+/*fa*^ male rats weighing 168–180 g were purchased from Harlan Laboratories (Charles River, Barcelona, Spain). The rats were housed in metabolic cages with a 12-h light-dark cycle and had free access to water and food. After 5 days of adaptation, 8 Zucker lean Lepr^+/*fa*^ and 8 Zucker-Lepr^*fa/fa*^ rats were euthanized as a reference (baseline). The remaining 40 Zucker-Lepr^*fa/fa*^ rats were then randomly assigned to receive 10^10^ colony-forming units (CFU) of one of the three probiotic strains, a mixture of *Lactobacillus paracasei* CNCM I-4034 and *Bifidobacterium breve* CNCM I-4035, or a placebo by oral gavage administration in a 0.5 ml volume as a single dose daily for 30 days. An additional group of 8 Zucker lean Lepr^+/*fa*^ rats received the placebo for 30 days (Fig. 1). The placebo contained 67% cow’s milk powder, 32.5% sucrose, and 0.56% vitamin C.

After the intervention, the animals were anesthetized and sedated with ketamine and xylazine. Blood was drawn from the aorta and centrifuged for 10 min at 1,000×*g* and 4 °C to separate the serum from the cells. Samples of intestinal mucosa were also taken.

### DNA microarray

Four animals per group were used in the DNA array. We used Affymetrix Rat Gene 1.1 ST Array Plates (Affymetrix Inc., Santa Clara, CA) following the manufacturer’s directions. Briefly, RNA extraction was performed using the RNeasy Mini Kit and Qiacube system. A quantity of 300 ng total RNA was utilized in cDNA synthesis with the Ambion WT Expression kit, and the resulting cDNA was fragmented using uracil-DNA glycosylase and APE1 (apurinic/apyrimidinic endonuclease-1). The labeling process was performed using the WT Terminal Labeling Kit (Affymetrix) with deoxynucleotidyl transferase linked to biotin. After fragmentation, 5.5 μg cDNA was hybridized using the GeneChip Hybridization, Wash and Stain Kit from Affymetrix. GeneChips were scanned using the GeneTitan™. The data were analyzed with the Command Console (AGCC 3.1, Affymetrix) and the Expression Console (EE 1.1, Affymetrix). The value definition was performed using the RMA (Robust Multichip Average) signal intensity.

**Figure 2:**
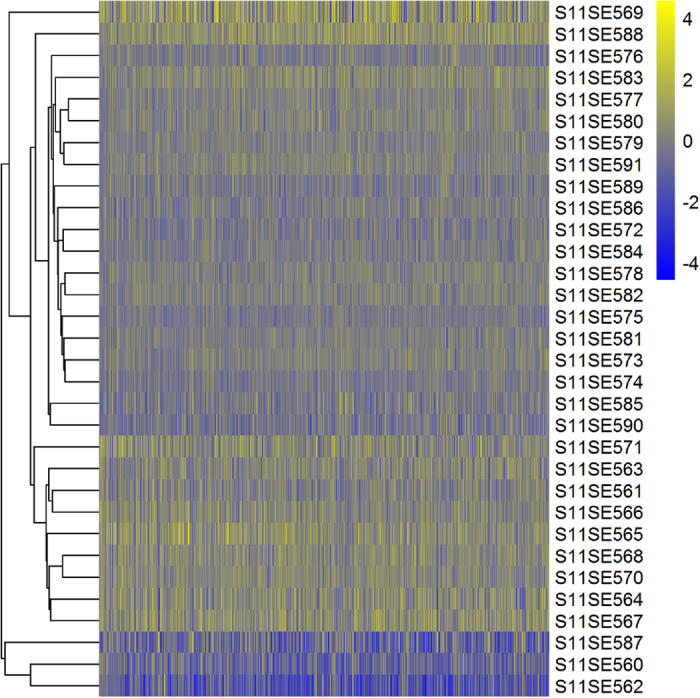
Microarray results obtained with the Rat Gene 1.1 ST Array Plate (Affymetrix®). These results were uploaded to the Gene Expression Omnibus (GEO) platform as ‘GSE73848 *Expression data from intestinal mucosa of Zucker rats*.’ *n*=4 rats per group.

The intensity value of each probe in the array analysis was normalized with the Robust Multichip Average (RMA) using the Partek Genomics Suite version 6.10 (Partek) to obtain an individual intensity for each set of probes. All expression data were averaged to achieve a unique expression value for the gene, and the background was deleted. The identification of expression changes was performed using multiple regression models comparing the intensity of each gene with the interaction (Zucker lean Lepr^*+/fa*^ or Zucker-Lepr^*fa/fa*^ rats that received placebo versus Zucker-Lepr^*fa/fa*^ rats that received any probiotic strain). The two-dimensional hierarchic cluster with the statistically significant sequences (*n*=936) appears in Fig. 2, according to the intensity and the aforementioned interactions.

## Data Records

The complete data set in the present study complies with the MIAME (Minimum Information About a Microarray Experiment) requirements and was uploaded into the *Gene Expression Omnibus* (GEO) database with the title *Expression data from intestinal mucosa of Zucker rats* and reference GSE73848 (Data Citation 1). Detailed information about each sample is in Table 1.

## Technical Validation

### Sample processing

Tissue samples were freeze-clamped in liquid nitrogen, and total RNA was extracted in an automatized fashion using the RNeasy Mini kit (Qiagen, Barcelona, Spain) in QIacube system (Qiagen). RNA quantity and quality were estimated in a Nanodrop and Bioanalyzer (NanoDrop Technologies, Winooski, Vermont, USA, and Agilent Genomics, Santa Clara, CA, USA, respectively).

### cDNA synthesis, fragmentation and labeling

cDNA was synthesized from 300 ng of total RNA using Ambion WT Expression kit (Thermo Fisher, Carlsbad, CA, USA), following the manufacturer’s directions. The main steps of the protocol were: cDNA synthesis with random primers that included the T7 promoter; *in vitro* transcription; new cDNA synthesis from the cRNA obtained in the previous step including dUTP. cDNA was fragmented with Uracil DNA Glycosylase (UDG) and Apurinic/apyrimidinic endonuclease 1 (AP1), and subsequently labeled using terminal transferase and WT Terminal Labeling kit (Affymetrix, Santa Clara, CA, USA).

**Figure 3:**
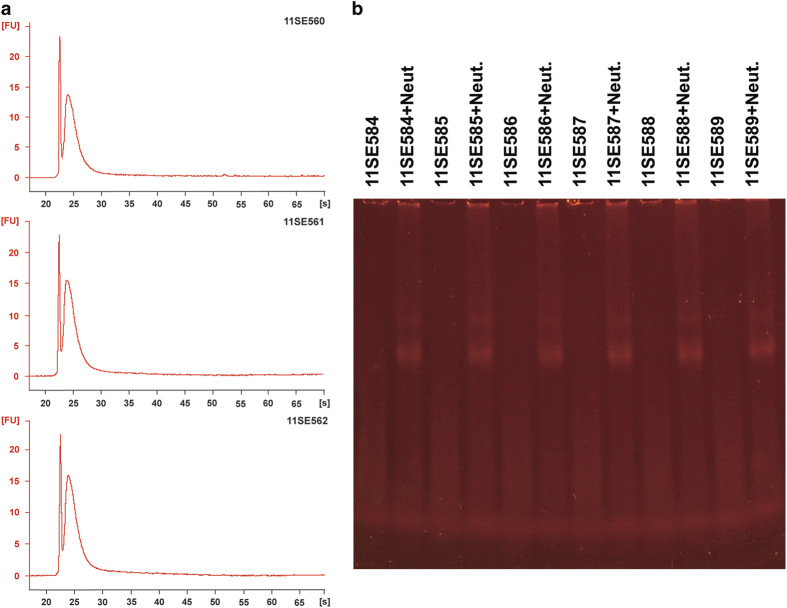
Fragmentation of cDNAs. (**a**) Bioanalyzer. (**b**) gel-shift assay.

An aliquot of each sample containing 5.5 μg of cDNA was fragmented and labeled with biotin using terminal transferase. The correct fragmentation was ensured by checking an aliquot in Bioanalyzer (Fig. 3a), and the correct labeling was checked by a gel-shift assay (Fig. 3b).

### Hybridization and scanning of GeneChip rat gene 1.1 ST array. Analysis of controls

**Figure 4:**
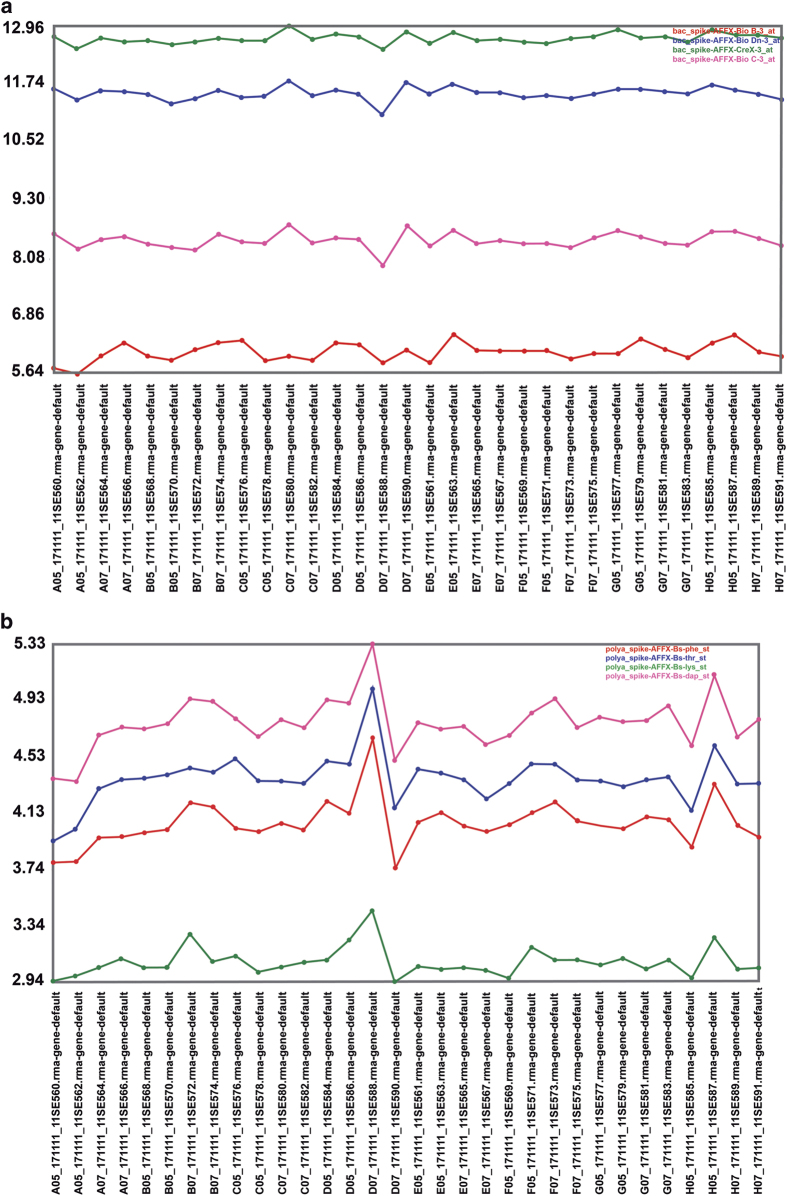
Quality control assessment for microarrays of the RNA samples from intestinal mucosa. (**a**) Hybridization controls. (**b**) Labeling controls with transcripts from *Bacillus subtilis*.

Three quality controls were used: hybridization, labeling and sample controls:

1. Hybridization (spike) controls were probes for sequences included in the hybridization mixture, and indicate that the hybridization, washing and development steps were correct. The spike controls used were BioB, BioC, BioD and Cre. Each was included in the mixture in increasing concentrations so BioB<BioC<BioD<Cre. Accordingly, the intensity of each probe must be proportional to the amount of oligo present in the mixture. The way to ensure that hybridization was correct (from the preparation of the hybridization mixture to the scanning) is to represent the intensity values of each probe in all samples, which must show increasing values, proportional to the concentration. The intensity values normalized by the spike controls in all samples appear in Fig. 4a. These values and the relationship among them were as expected. The absolute values are not as important as the general trend, that is, the lines must not cross.

2. Labeling controls were polyA controls. These were transcripts from *Bacillus subtilis* added in increasing concentrations to the RNA of the sample during the processing. The intensity values of each polyA control in each sample allows to check whether cDNAcRNA synthesis was correct. As was the case for spike controls, intensity values of each probe in all samples were plotted and showed an increasing trend proportional to the concentrations. The intensity values normalized for polyA controls in each of the samples analyzed appear in Fig. 4b.

In this plot, an increase in the intensity of each polyA control is expected to occur as a function of the concentration. The absolute value is not as important as the general trend, that is, the lines must not cross one another. In our experiment the intensity values and the relationship among them were as expected.

3. Sample controls allowed the identification of outliers, that is, samples with a behavior completely different from the rest. The parameters analyzed in this study were:

- Pos_vs_neg_auc: it is a measure of the detection of the positive controls versus the false detection of the negative controls. It is a robust measure of the global quality of the data. Typical values range 0.8–0.9, being 1.0 the perfect value. In contrast, 0.5 indicates a lack of difference between positive and negative controls. Values <0.8 indicate that the sample may be an outlier. All of our samples were acceptable as shown in Table 2.

- All Probe set Mean: it is a measure of the average signal of all probe sets included in the analysis. It allows the identification of arrays too bright or too dark. As shown in Table 2, none of the samples was an outlier.

- All Probe Set RLE Mean: the signal from each probe set is compared with the average signal of that probe set in the study. This parameter is the average of those differences for all the probe sets. Generally, high values indicate that the signals in an array are very different from the rest of the signals of the study. In this kind of array, values usually range 0.27–0.61 for studies that include samples from various tissues, and 0.1–0.23 for technical replicates. Our values were consistent (Table 2).

### Bioinformatic analysis

Gene expression results obtained from 32 RNA samples were analyzed. The chip used was RatGene1.1ST array plate (Affymetrix, Santa Clara, CA, USA). The goal of the analysis was to obtain sequences differentially expressed under our experimental conditions. The bioinformatic analysis contained the following steps:

a) Quality control of the arrays.

b) Pre-processing of data by the RMA method.

c) Normalization of arrays to obtain the worklist.

All these steps are described in more detail below.

### Analysis of outliers

The quality of the arrays was ensured with the following controls:

Array Outlier: indicates the percentage of transcripts whose levels in the array are inconsistent with the levels in the rest of arrays of the experiment. The software used, dChip^14^, recommends to take with precaution those samples that reach a value >5%, and eliminate those that reach a value >15%. According to these criteria, sample 11SE562 must be handled with precaution and its behavior will be carefully studied in the following steps (Table 3).

**Figure 5:**
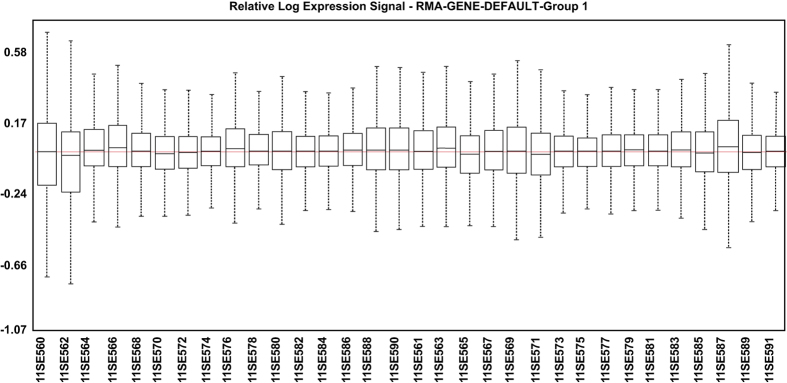
Relative log expression signal graph. This reference array is made from the median of all the arrays for each probe set. It is assumed that most of the expression values do not change with respect to the median and, accordingly, are around 0.

Relative Log Expression (RLE): Expression Console software (Affymetrix) was used to obtain RLE values in which the expression value of each probe set of each sample was normalized by a reference array. This reference array is made from the median of all the arrays for each probe set. It is assumed that most of the expression values do not change with respect to the median and, accordingly, are around 0. All samples exhibited correct RLE values (Fig. 5).

### Pre-processing of data

The intensity values of each probe were processed and normalized by the Robust Multichip Average (RMA) method to obtain an individual intensity value for each set of probes. Subsequently, all expression values of each exon were averaged to obtain unique expression values per gene to get a list of 29,214 probe sets.

### First filtering of data

Data from GeneChips were filtered to discard those sequences with hybridization signals close to background. The number of sequences that passed the filtering was 10,015.

### Normalization of data

The array data saved in the *.cel files were subjected to a normalization step by quantiles using the Partek Genomics Suite software (v6.10).

### Second filtering of data

Finally, the 10,015 sequences were filtered to discard those probes that did not show any changes in expression in all samples, based on the standard deviation of the normalized intensity data. The value used as a limit of expression change among conditions allows the elimination of those sequences that do not vary in the comparison among any sample and therefore are not informative. This filter narrowed the sequences down to 6,990.

## Additional information

**How to cite this article:** Plaza-Díaz, J. *et al.* Gene expression profiling in the intestinal mucosa of obese rats administered probiotic bacteria. *Sci. Data* 4:170186 doi: 10.1038/sdata.2017.186 (2017).

**Publisher’s note:** Springer Nature remains neutral with regard to jurisdictional claims in published maps and institutional affiliations.

## Supplementary Material



## Figures and Tables

**Table 1:** Data description of ‘Expression data from intestinal mucosa of Zucker rats’.

**GEO-ID**	**organism**	**tissue**	**Sample description**	**analysis**
GSM1904446	*Rattus norvegicus*	intestinal mucosa	ZL reference	Microarray
GSM1904447	*Rattus norvegicus*	intestinal mucosa	ZL reference	Microarray
GSM1904448	*Rattus norvegicus*	intestinal mucosa	ZO reference	Microarray
GSM1904449	*Rattus norvegicus*	intestinal mucosa	ZO reference	Microarray
GSM1904450	*Rattus norvegicus*	intestinal mucosa	ZL placebo	Microarray
GSM1904451	*Rattus norvegicus*	intestinal mucosa	ZL placebo	Microarray
GSM1904452	*Rattus norvegicus*	intestinal mucosa	ZO placebo	Microarray
GSM1904453	*Rattus norvegicus*	intestinal mucosa	ZO placebo	Microarray
GSM1904454	*Rattus norvegicus*	intestinal mucosa	ZO *B. breve* CNCM I-4035 and *L. paracasei* CNCM I-4034 rat 1	Microarray
GSM1904455	*Rattus norvegicus*	intestinal mucosa	ZO *B. breve* CNCM I-4035 and *L. paracasei* CNCM I-4034 rat 2	Microarray
GSM1904456	*Rattus norvegicus*	intestinal mucosa	ZO *B. breve* CNCM I-4035 rat 1	Microarray
GSM1904457	*Rattus norvegicus*	intestinal mucosa	ZO plus *B. breve* CNCM I-4035 rat 2	Microarray
GSM1904458	*Rattus norvegicus*	intestinal mucosa	ZO plus *L. paracasei* CNCM I-4034 rat 1	Microarray
GSM1904459	*Rattus norvegicus*	intestinal mucosa	ZO plus *L. paracasei* CNCM I-4034 rat 2	Microarray
GSM1904460	*Rattus norvegicus*	intestinal mucosa	ZO plus *L. rhamnosus* CNCM I-4036 rat 1	Microarray
GSM1904461	*Rattus norvegicus*	intestinal mucosa	ZO plus *L. rhamnosus* CNCM I-4036 rat 2	Microarray
GSM1904462	*Rattus norvegicus*	intestinal mucosa	ZL reference	Microarray
GSM1904463	*Rattus norvegicus*	intestinal mucosa	ZL reference	Microarray
GSM1904464	*Rattus norvegicus*	intestinal mucosa	ZO reference	Microarray
GSM1904465	*Rattus norvegicus*	intestinal mucosa	ZO reference	Microarray
GSM1904466	*Rattus norvegicus*	intestinal mucosa	ZL placebo	Microarray
GSM1904467	*Rattus norvegicus*	intestinal mucosa	ZL placebo	Microarray
GSM1904468	*Rattus norvegicus*	intestinal mucosa	ZO placebo	Microarray
GSM1904469	*Rattus norvegicus*	intestinal mucosa	ZO placebo	Microarray
GSM1904470	*Rattus norvegicus*	intestinal mucosa	ZO *B. breve* CNCM I-4035 and *L. paracasei* CNCM I-4034 rat 3	Microarray
GSM1904471	*Rattus norvegicus*	intestinal mucosa	ZO plus *B. breve* CNCM I-4035 and *L. paracasei* CNCM I-4034 rat 4	Microarray
GSM1904472	*Rattus norvegicus*	intestinal mucosa	ZO plus *B. breve* CNCM I-4035 rat 3	Microarray
GSM1904473	*Rattus norvegicus*	intestinal mucosa	ZO plus *B. breve* CNCM I-4035 rat 4	Microarray
GSM1904474	*Rattus norvegicus*	intestinal mucosa	ZO plus *L. paracasei* CNCM I-4034 rat 3	Microarray
GSM1904475	*Rattus norvegicus*	intestinal mucosa	ZO plus *L. paracasei* CNCM I-4034 rat 4	Microarray
GSM1904476	*Rattus norvegicus*	intestinal mucosa	ZO plus *L. rhamnosus* CNCM I-4036 rat 3	Microarray
GSM1904477	*Rattus norvegicus*	intestinal mucosa	ZO plus *L. rhamnosus* CNCM I-4036 rat 4	Microarray
Abbreviations: ZL, Zucker lean Lepr^+*/fa*^ rats; ZO, Zucker Lepr*^fa/fa^* rats. *n*=4 rats per group.				

**Table 2:** Array outliers.

**Sample**	**pos_vs_neg_auc**	**all_probeset_mean**	**all_probeset_rle_mean**	**Sample**	**pos_vs_neg_auc**	**all_probeset_mean**	**all_probeset_rle_mean**
11SE560	0.85422	5.541424	0.241511	11SE576	0.864426	5.589662	0.15491
11SE561	0.878029	5.57988	0.171301	11SE577	0.871134	5.576477	0.129484
11SE562	0.865219	5.507742	0.248986	11SE578	0.88545	5.576419	0.128479
11SE563	0.864947	5.589251	0.175722	11SE579	0.866908	5.576636	0.127985
11SE564	0.864352	5.5837	0.173877	11SE580	0.857355	5.586262	0.153071
11SE565	0.877761	5.56282	0.161048	11SE581	0.867117	5.574453	0.131372
11SE566	0.871477	5.586922	0.175144	11SE582	0.88222	5.572265	0.130127
11SE567	0.877053	5.580364	0.167735	11SE583	0.874066	5.581122	0.141788
11SE568	0.860977	5.579972	0.140385	11SE584	0.880905	5.571299	0.122088
11SE569	0.880092	5.582358	0.217311	11SE585	0.846603	5.579253	0.170314
11SE570	0.882	5.574604	0.148582	11SE586	0.876601	5.576181	0.131724
11SE571	0.888591	5.56232	0.194358	11SE587	0.872058	5.566449	0.226278
11SE572	0.876405	5.570266	0.133905	11SE588	0.904137	5.559667	0.184642
11SE573	0.879322	5.56958	0.12135	11SE589	0.873042	5.570007	0.150338
11SE574	0.875899	5.572968	0.116654	11SE590	0.842198	5.593665	0.170785
11SE575	0.863548	5.58026	0.129136	11SE591	0.879472	5.567289	0.125395
Array outliers. **Pos_vs_neg_auc** is a measure of the detection of the positive controls versus false detection of the negative controls. It is a robust measure of the global quality of the data. Typical values range 0.8–0.9, being 1.0 the perfect value. In contrast, 0.5 indicates a lack of difference between positive and negative controls. Values <0.8 indicate that the sample may be an outlier. **All Probe set Mean** is a measure of the average signal of all probe sets included in the analysis. It allows the identification of arrays too bright or too dark. **All Probe Set RLE Mean:** the signal from each probe set is compared with the average signal of that probe set in the study. This parameter is the average of those differences for all the probe sets. Generally, high values indicate that the signals in an array are very different from the rest of the signals of the study. In this kind of array, values usually range 0.27–0.61 for studies that include samples from various tissues, and 0.1–0.23 for technical replicates. Abbreviations: RLE, relative log expression.							

**Table 3:** Array quality control percentage.

**Sample**	**% Array outlier**	**Sample**	**% Array outlier**
11SE560	3.305	11SE576	0.23
11SE561	0.271	11SE577	0.254
11SE562	6.853	11SE578	0.274
11SE563	0.192	11SE579	0.237
11SE564	0.394	11SE580	0.405
11SE565	0.329	11SE581	0.281
11SE566	0.291	11SE582	0.254
11SE567	0.339	11SE583	0.298
11SE568	0.312	11SE584	0.35
11SE569	0.668	11SE585	0.422
11SE570	0.312	11SE586	0.35
11SE571	0.35	11SE587	0.405
11SE572	0.302	11SE588	0.566
11SE573	0.298	11SE589	0.291
11SE574	0.288	11SE590	0.247
11SE575	0.247	11SE591	0.226
Percentage of transcripts whose levels in the array are inconsistent with the levels in the rest of arrays of the experiment.			
